# Surface Roughening of Pt-Polystyrene Spherical Janus Micromotors for Enhanced Motion Speed

**DOI:** 10.3390/mi13040555

**Published:** 2022-03-31

**Authors:** Le Zhou, Yi Wei, Hongwen Zhang, Zhulin Huang, Shuyi Zhu, Zhipeng Zhao, Yujing Guo, Hao Fu, Qian Zhao, Weiping Cai

**Affiliations:** 1 Key Lab of Materials Physics, Anhui Key Lab of Nanomaterials and Nanotechnology, Institute of Solid State Physics, HFIPS, Chinese Academy of Sciences, Hefei 230031, China; zhoule@issp.ac.cn (L.Z.); yiwei83@issp.ac.cn (Y.W.); zhushuyi@issp.ac.cn (S.Z.); zzp13@mail.ustc.edu.cn (Z.Z.); yjguo@issp.ac.cn (Y.G.); haofu@issp.ac.cn (H.F.); zhaoqian@issp.ac.cn (Q.Z.); 2 Science Island Branch of Graduate School, University of Science and Technology of China, Hefei 230026, China

**Keywords:** Pt-PS micromotors, plasma etching, surface roughening, Pt-pushed motion, speed enhancement

## Abstract

Spherical Janus micromotors (SJMs) have attracted much attention, and their high-speed motion is highly desired due to their various potential applications. However, the conventional template-deposition method often leads to an active Pt coating with a smooth surface, which is unbeneficial to speed enhancement in terms of catalytic reaction. Here, a facile surface roughening method is presented to fabricate the Pt-polystyrene (PS) SJMs with rough Pt surface (or Pt_r_-PS SJMs) by plasma-etching the PS colloidal monolayer and then depositing Pt. The Pt_r_-PS SJMs can exhibit directional motion pushed by the Pt in the various H_2_O_2_ solutions, and they show much higher motion speeds than the Pt-PS SJMs with smooth Pt surfaces at the same H_2_O_2_ concentration. The Pt-pushed motion is related to the locally asymmetric catalytic reaction of the Pt coating on PS. The speed is also associated with the surface roughness of the Pt coating. The Pt film with a rough surface causes enhanced motion speed due to the improvement of reaction catalytic activity. This work presents a new route to enhancing the motor motion speed, which is of significance in designing micromotors with high-speed motion.

## 1. Introduction

Micromotors (or colloidal motors) are capable of self-propulsion in fluid by absorbing surrounding external energy [1,2,3]. A typical example is chemically powered micromotors, which can propel themselves in a fuel (i.e., H_2_O_2_)-containing solution by chemical reaction [4,5,6]. Recently, more attention has been paid to these micromotors due to their wonderful potential applications in the fields of environmental science and biomedicine [7,8,9,10].

Notably, the present types of micromotors mainly include spherical Janus micromotors (SJMs) [11,12,13,14,15,16,17,18], and rod-like [19,20,21,22] and tubular [23,24,25,26] micromotors. Among them, the SJMs attract the most attention, probably due to their easy fabrication. The typical fabrication procedure includes spreading the dispersions of the nearly smooth spherical particles, such as polystyrene (PS) (or other polymers) [13,14], SiO_2_ [15,16], and TiO_2_ [18] microspheres, on the substrate (silicon wafer or glass slides) to form a colloidal monolayer, and subsequent physical deposition of the active composition (e.g., Pt) to obtain half-coated SJMs [27]. Accordingly, the resulting SJMs usually have a smooth surface for active coating, which is unbeneficial for improving their catalytic reaction ability.

The motor catalytic reaction, except for fuel concentration, can be dominated by the morphological characteristics of the active catalytic coating [28,29]. The rough outer surface can promote the reaction. For instance, Wang et al. [28] focused on the preparation of Pt-PS Janus dimers with rough Pt surface by chemical route and found that these dimers could exhibit significant motion speed enhancement and varied motion mechanisms in an H_2_O_2_-contained solution, compared to Pt-PS motors prepared by physical deposition. Wu et al. [29] coated polymer capsules with a few dendritic Pt nanoparticles (NPs) to form Janus motors, showing the enhanced ability of catalytic decomposition and the resulting speed enhancement. However, little effort has been made to improve the motion speed of these common SJMs.

Here, we present a facile surface roughening method to fabricate Pt-PS SJMs with rough Pt surfaces (or the Pt_r_-PS SJMs) by plasma-etching the PS colloidal monolayer and then depositing Pt. The Pt-PS SJMs can exhibit directional motion, pushed by the Pt in different H_2_O_2_ solutions, and the Pt_r_-PS SJMs show much higher speed than that of the conventionally prepared Pt-PS SJMs with smooth Pt surfaces (or Pt_s_-PS SJMs) at the same H_2_O_2_ concentration. The rough surface can improve the reaction of catalytic activity, leading to motion speed enhancement. This work can provide a new route to enhancing the motor motion speed for the SJMs, which is of significance in designing micromotors with high-speed motion.

## 2. Experimental Section

### 2.1. Materials and Chemicals

Polystyrene (PS) microsphere (ϕ2 µm) suspension was purchased from Shanghai Huge Biotechnology Co., Ltd. (Shanghai, China). High-purity SF_6_ gas (99.999%) and Pt target (ϕ50 × 1.0 mm) were obtained from Hefei Ningte Gas Management Co., Ltd. (Hefei, China), and Hefei Kejing Materials Technology Co., Ltd. (Hefei, China), respectively. Ethanol and H_2_O_2_ were bought from Sinopharm Chemical Reagent Co., Ltd. (Shanghai, China). The silicon wafer and glass pieces were obtained from Hefei Baierdi Chemical Technology Co., Ltd. (Hefei, China). Deionized water with a resistivity of 18.2 MΩ cm at 25 °C was used to prepare the aqueous solution.

### 2.2. Fabrication of the Pt_r_-PS SJMs

**Scheme 1:**
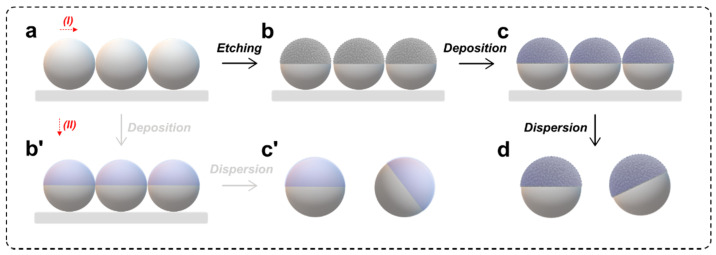
The schematic illustration of the routes of (I) the Pt_r_-PS SJMs and (II) the Pt_s_-PS SJMs. Route (I): (**a**) The self-assembled PS colloidal monolayer; (**b**) the etched PS colloidal monolayer; (**c**) the Pt_r_-PS colloidal monolayer; (**d**) The dispersed Pt_r_-PS SJMs. Route (II): (**b’**) The Pt_s_-PS colloidal monolayer; (**c’**) The dispersed Pt_s_-PS SJMs.

The Pt_r_-PS SJMs were fabricated by depositing Pt on an etched PS colloidal monolayer, as illustrated in Scheme 1. This process included the preparation of PS colloidal monolayer (Scheme 1a), etching (Scheme 1b), sputtering deposition, and dispersion (Scheme 1c,d).

#### 2.2.1. Preparation and Etching of the PS Colloidal Monolayer

The PS colloidal monolayer was prepared by the interface self-assembly method according to previous reports [30,31,32]. A glass piece was ozone-cleaned for 20 min and covered by a thin layer of water membrane. The PS-contained suspension was diluted with the same volume of ethanol, and then slowly injected into the water membrane until a complete white film was formed. Afterward, the film was transferred onto the silicon piece (1 cm × 1 cm) to obtain a PS colloidal monolayer (Scheme 1a).

Surface roughening of the PS colloidal monolayer was achieved by SF_6_ plasma etching (Scheme 1b) [33]. Typically, the monolayer was placed in a home-built etching machine and etched by SF_6_ plasma for 2 min, during which the vacuum degree, volume flowrate, and power were maintained at 10 Pa, 50 sccm, and 200 W, respectively.

#### 2.2.2. Preparation of the Pt_r_-PS SJMs

The etched PS monolayer was then deposited with Pt in a sputtering deposition apparatus (Quorum Q150R S Plus), as shown in Scheme 1c. During the deposition, the vacuum degree, sputtering current, and deposition rate were maintained at 0.03 mbar, 30 mA, and 10 nm/min, respectively. After depositing for 2 min, a Pt deposition layer with a thickness around 20 nm was formed. Finally, the Pt_r_-PS SJMs dispersed in 2 mL water were obtained by peeling and ultrasonic dispersion for 30 s (Scheme 1d).

For comparison, the Pt_s_-PS SJMs were prepared by directly sputtering a 20 nm Pt coating on the un-etched PS colloidal monolayer (Scheme 1b’,c’).

### 2.3. Characterizations and Motion Observations

A field emission scanning electron microscope (FESEM, SU8020, Hitachi, Tokyo, Japan) attached to an Oxford energy dispersion spectrometer (EDS) was used to characterize the morphology and composition. The surface roughness information was captured by an atomic force microscope (AFM, NX10, Park, Suwon, Korea). For all Pt-PS SJMs, their motion in the solution was observed by an inverted optical microscope (IX73, Olympus, Tokyo, Japan) and recorded into the motion videos. The solution samples were prepared by mixing the same volume of SJMs-contained aqueous solution and the H_2_O_2_ solution with different concentrations (from 0 to 30 wt%). During the observations, the samples were loaded by a thin coverslip. For motion analysis, the SJM motion in the videos was tracked as a pixel value by the software. Thereafter, the real motion information was converted from the scale bar with its pixel value, and further estimated via the corresponding physical definitions. Motion analysis of the SJMs counted at least four SJMs.

## 3. Results and Discussion

**Figure 1:**
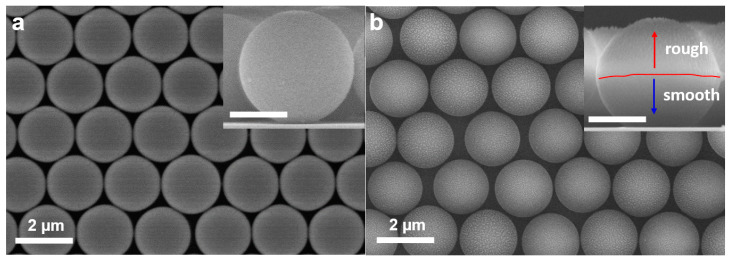
FESEM image of the PS colloidal monolayer (**a**) before and (**b**) after ion etching treatment. Insets show corresponding enlarged side views. Scale bars are both 1 micron.

First, after the interface self-assembly, a PS colloidal monolayer built of the nearly close-packed PS microspheres with a smooth surface was formed, as shown in Figure 1a. Then, an etched PS colloidal monolayer was obtained after the plasma etching, as shown in Figure 1b. Compared with the original monolayer, the etched monolayer became slightly loose, and each PS microsphere in the monolayer exhibited an obviously rougher surface. Especially, it is relatively obvious from the side view that the grooves produced by etching were left, leading to surface roughening.

### 3.1. Morphology and Structure

**Figure 2:**
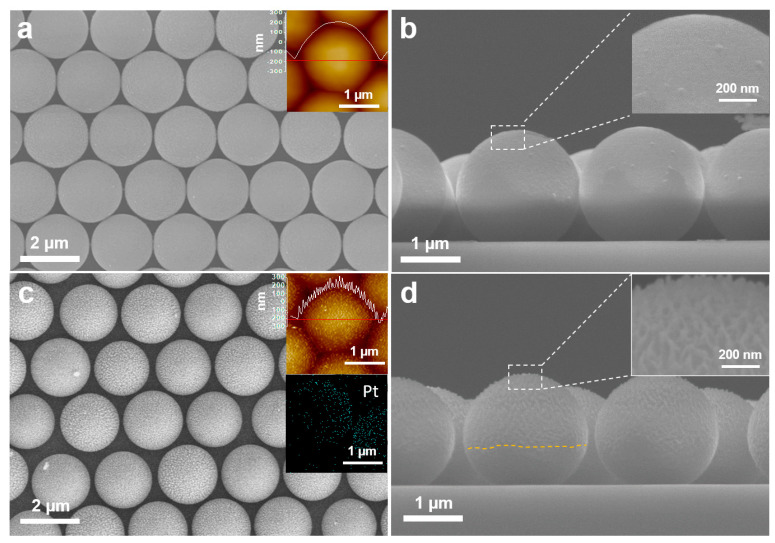
FESEM image of the Pt-PS SJMs obtained by sputtering Pt onto PS spheres (**a**,**b**) without and (**c**,**d**) with etching treatment. (**a**,**c**) Top view. (**b**,**d**) Side view. AFM examinations for roughness analysis, enlarged SEM for detailed surface observation and Pt elemental analysis are shown in insets.

The Pt-PS SJMs were prepared after the Pt sputtering deposition, and the corresponding characterizations are shown in Figure 2. In addition, the corresponding surface roughness was analyzed by AFM examination. It demonstrates that, for PS spheres without etching treatment, a smooth Pt layer is obtained, while those after etching treatment possess a rough Pt surface, with nanoscale height fluctuations (the white curve in up-right inset of Figure 2c) along the surface profile (the red line in down-right inset of Figure 2c) of a PS microsphere, obtaining the Pt_r_-PS SJMs. This obvious difference can be further confirmed by the SEM side view, in which obvious dividing lines (the yellow dot curve in Figure 2d) in the middles of the PS microspheres and their cap-like coatings on the upper surface are extremely rough or rugged. In addition, the elemental analysis of Pt_r_-PS SJMs illustrates that the coating of Pt covers only the top half of the spheres, achieving a dissymmetrical structure (see down-right inset in Figure 2c).

Briefly, based on an additional plasma etching, the obtained Pt_r_-PS SJMs exhibit obviously rougher surfaces than the Pt_s_-PS SJMs. The formation of such a rough surface is closely associated with the etching and deposition process. The grooves (or small particles) are formed first during the etching on the surface of the PS microspheres due to the disordered etching reaction [33], and then the nearly conformal deposition of the Pt coating caused by slow deposition rate results in the final rough surface.

### 3.2. Self-Propelled Behavior

**Figure 3:**
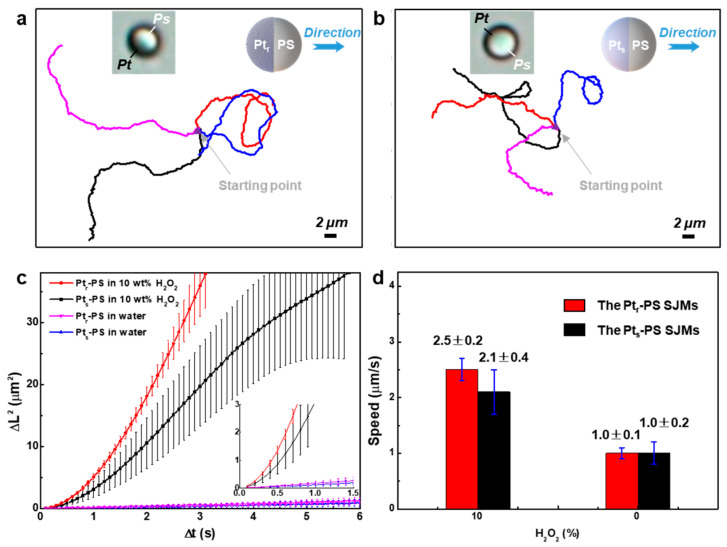
(**a**,**b**) The trajectories obtained from the motion videos and shifted to the same starting point, corresponding to the different (**a**) Pt_r_-PS SJMs and (**b**) Pt_s_-PS SJMs moving in 10 wt% H_2_O_2_ within 12 s. The top insets in each figure are the corresponding optical images of a typical SJM and a schematic illustration of the motion direction. (**c**) MSD curves. (**d**) Average speed.

Next, taking H_2_O_2_ as fuel, we observed the motion behavior of the Pt_r_-PS SJMs in a fuel-containing aqueous solution. It can be seen clearly under an optical microscope that the spherical particles built of dark side (or Pt) and white body (or PS) are Pt_r_-PS SJMs, as typically shown in the up-left inset of Figure 3a. Without exception, the Pt_r_-PS SJMs exhibit obviously directional movement as they are pushed by the Pt when immersed in 10 wt% H_2_O_2_ solution, as shown in Video S1 and Figure 3a. Additionally, the slight ‘oscillation movement’ (referring to suddenly changed speed, such as obvious pause or acceleration) can be observed intermittently during the motion process (see the Video S1). Simultaneously, the mean square displacement (MSD) curve (the red curve in Figure 3c) is parabola-like, especially at short time intervals, and exhibits large motion displacement, consistent with the directional self-propelled motion [14,15,34]. Contrarily, they only show Brownian movement (see Video S2a) in a small space, with obviously irregular motion trajectories (see Figure S1a), reflected on the MSD curve as a nearly straight line (the magenta curve in Figure 3c) [14,15,34].

For comparison, the Pt_s_-PS SJMs show similar directional motion to the Pt_r_-PS SJMs, exhibiting directional motion as they are pushed by the Pt in the H_2_O_2_-contained solution (Video S3 or Figure 3b,c), whereas they show irregular Brownian movement in water (Video S2b, Figure S1b, or Figure 3c). However, the more obviously intermittent oscillation (Video S3) and slower motion speed from the perspectives of trajectories and MSD curves (Figure 3b,c) can be observed and proved.

More intuitively, the average motion speed was estimated according to the actual motion trajectories (or distance) with the spent time, as shown in Figure 3d. All the SJMs’ speed is increased in the H_2_O_2_-contained solution, but compared with the average speed of 2.1 µm/s for the Pt_s_-PS SJMs, the speed of the Pt_r_-PS SJMs is up to 2.5 µm/s, achieving a speed enhancement of 19%.

**Figure 4:**
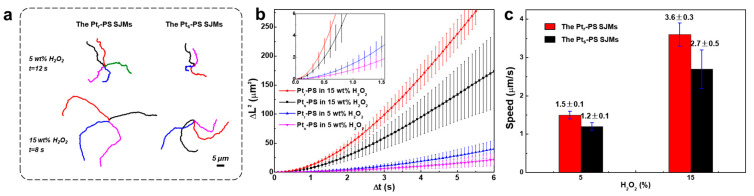
(**a**) The integrated trajectories obtained from the motion videos for all SJMs moving in 5 wt% H_2_O_2_ within 12 s and 15 wt% H_2_O_2_ within 8 s, respectively. (**b**) MSD curves. The inset is the enlarged plot of the short-time-interval regime. (**c**) Average speed.

Further, if the H_2_O_2_ concentration was reducing (or increasing) to 5 wt% (or 15 wt%), the directional self-propulsion, pushed by the Pt, could remain unchanged for the Pt_r_-PS SJMs, as shown in Videos S4 and S5 or Figure 4a,b. Notably, the oscillation motion is more obvious in the low concentration of (or 5 wt%) H_2_O_2_ (see Video S4), whereas the nearly smooth motion will be easily observed in the high concentration of (or 15 wt%) H_2_O_2_ (see Video S5). By contrast, the Pt_s_-PS SJMs also show similar behavior (see Videos S6 and S7 or Figure 4a,b), but they still exhibit generally shorter trajectories (or displacement) than the Pt_r_-PS SJMs at the same spent time, showing the same phenomenon in the 10 wt% H_2_O_2_.

Eventually, the average speed was calculated, as given in Figure 4c. The speed for the Pt_r_-PS and Pt_s_-PS SJMs is 1.5 and 1.2 µm/s in 5 wt% H_2_O_2_ solution, as well as 3.6 and 2.7 µm/s in 15 wt% H_2_O_2_ solution, respectively. In both cases, the speed enhancement of the Pt_r_-PS SJMs is 25% and 33%, respectively. Obviously, the speed of the Pt_r_-PS SJMs can be generally enhanced at various H_2_O_2_ concentrations. Especially at a higher H_2_O_2_ concentration, such enhancement is more significant.

### 3.3. Rough Pt Surface-Enhanced Self-Propelled Mechanism

The Pt composition for the SJMs is the only catalyst to decompose the H_2_O_2_ and no bubbles can be observed around them when moving. Hence, the diffusiophoresis mechanism will play a major role in their self-propulsion.

**Figure 5:**
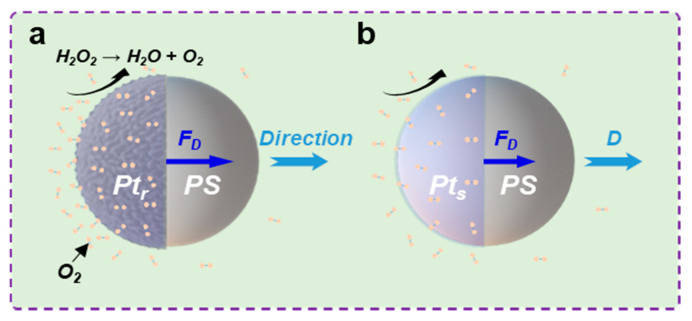
Schematic illustration of the driving mechanism for different Pt-PS SJMs. (**a**) The Pt_r_-PS SJMs; (**b**) The Pt_s_-PS SJMs.

As illustrated in Figure 5, for both kinds of SJMs, Pt coating is asymmetrically distributed on one side and will locally decompose H_2_O_2_ into O_2_ and H_2_O molecules. Due to the continuous reaction, the localized high molecule concentration (especially O_2_ concentration) is immediately formed and then diffused around, which produces a large force with the SJMs’ local surface through a collision when they meet. Such force is the main driving force (or F_D_) that promotes the directional self-propulsion away from the Pt, as reported previously [13,14,15].

Differently, the Pt_r_-PS SJMs always exhibit a faster speed than the Pt_s_-PS SJMs in the same H_2_O_2_-contained solution, which is closely related to their rougher Pt surface. This is because in terms of the catalytic reaction, the former type of SJM (see Figure 5a) could usually show a more violent chemical reaction than the latter type of SJM (see Figure 5b) due to the increased surface area and the enhanced reaction activity, beneficial for the non-dense tiny particles on the rough surface, resulting in enhanced motion speed, as we observed (see Figure 3 and Figure 4).

However, except for the catalyst, the chemical reaction is also dominated by reactant concentration as well as mass transport, as in our case. In a low concentration of H_2_O_2_ (say, 5 wt%), the reaction might be subject to insufficient H_2_O_2_ concentration. At this moment, the H_2_O_2_ surrounding the SJMs could be consumed rapidly, whereas its effective diffusion to the SJMs’ surface would take more time, which could be exactly reflected from the oscillation motion (Video S4 or S6) at low H_2_O_2_ concentration. In other words, a cumulative time was needed for the chemical reaction. Accordingly, the catalysts with different surface areas may have limited effects on the reaction at the low H_2_O_2_ concentration, leading to little speed enhancement. Conversely, in a high concentration of H_2_O_2_ (say, 15 wt%), the reaction might be subject to the surface of the catalyst. At this time, the H_2_O_2_ could be adequate, and the different Pt surface would have obviously different reaction effects. The rough Pt could thus show a faster reaction and, therefore, an obviously higher speed, as shown in Figure 5.

## 4. Conclusions

In summary, we have presented a facile surface roughening method to fabricate Pt_r_-PS SJMs by plasma-etching a PS colloidal monolayer and then depositing Pt. The Pt coating of the obtained Pt_r_-PS SJMs is much rougher than that of the Pt_s_-PS SJMs. It was found that the Pt-PS SJMs can exhibit directional motion away from the Pt when immersed in various H_2_O_2_ solutions. However, the Pt_r_-PS SJMs always show an enhanced speed compared to Pt_s_-PS SJMs at the same H_2_O_2_ concentration. The Pt-pushed directional self-propulsion is due to a local catalytic reaction of the Pt coating with an asymmetric distribution, which produces the directional driving force. The speed enhancement is mainly due to the rough Pt surface-enhanced reaction rate in terms of increasing reactive surface area and activity, which could be more significant at high H_2_O_2_ concentrations. This work provides a new route to enhancing the motor motion speed, and it is of significance in the design of micromotors with high-speed movement.

## Supplementary Materials

The following supporting information can be downloaded at: https://www.mdpi.com/article/10.3390/mi13040555/s1, Figure S1: Optical images captured from the videos of (a) the Pt_r_-PS SJMs and (b) the Pt_s_-PS SJMs moving in water within 12 s; Video S1: The motion video of the Pt_r_-PS SJMs moving in 10 wt% H_2_O_2_ with 12 s; Video S2: The motion video of the (a) Pt_r_-PS and (b) Pt_s_-PS SJMs moving in water with 12 s; Video S3: The motion video of the Pt_s_-PS SJMs moving in 10 wt% H_2_O_2_ with 12 s; Video S4: The motion video of the Pt_r_-PS SJMs moving in 5 wt% H_2_O_2_ with 12 s; Video S5: The motion video of the Pt_r_-PS SJMs moving in 15 wt% H_2_O_2_ with 8 s; Video S6: The motion video of the Pt_s_-PS SJMs moving in 5 wt% H_2_O_2_ with 12 s; Video S7: The motion video of the Pt_s_-PS SJMs moving in 15 wt% H_2_O_2_ with 8 s.
