# Supplementary material for: Surface Roughening of Pt-Polystyrene Spherical Janus Micromotors for Enhanced Motion Speed
Source: Micromachines (Basel). 2022 Mar 31;13(4):555. doi: 10.3390/mi13040555 (PMC9029801; doi:10.3390/mi13040555)
Supplement: Supplementary file 1 [file micromachines-13-00555-s001.zip › Figure S1.pdf]

Surface Roughening of Pt-Polystyrene Spherical Janus Micro-motors for Enhanced Motion Speed

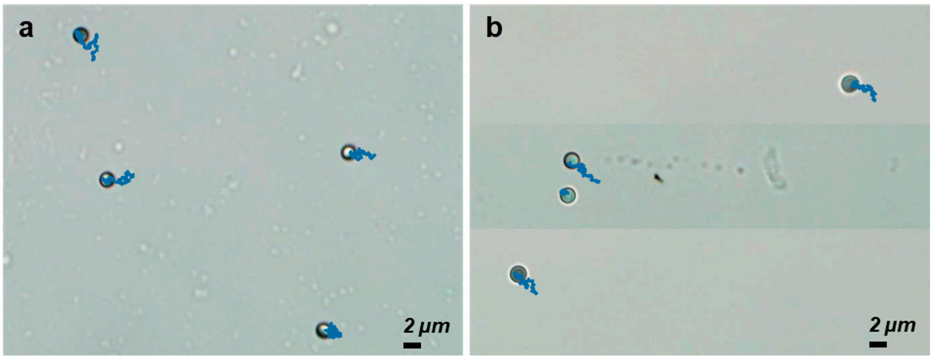

Figure S1. Optical images captured from the videos of (a) the Pt_r-PS SJMs and (b) the Pt_s-PS SJMs moving in water within 12 s. The blue curves represent the corresponding motion trajectories.
